# Vacuum-assisted mini-percutaneous nephrolithotomy is associated with lower rates of infectious complications compared to vacuum-cleaner procedure in patients at high risk for infections: a single-center experience

**DOI:** 10.1007/s00345-024-04897-3

**Published:** 2024-03-27

**Authors:** Andrea Marmiroli, Marco Nizzardo, Stefano Paolo Zanetti, Gianpaolo Lucignani, Matteo Turetti, Carlo Silvani, Franco Gadda, Fabrizio Longo, Elisa De Lorenzis, Giancarlo Albo, Andrea Salonia, Emanuele Montanari, Luca Boeri

**Affiliations:** 1 Department of Urology, Fondazione IRCCS Ca’ GrandaOspedale Maggiore Policlinico, Milan, Italy; 2 Department of Clinical Sciences and Community Health, University of Milan, Milan, Italy; 3 Division of Experimental Oncology/Unit of Urology, URI, IRCCS Ospedale San Raffaele, University Vita-Salute San Raffaele, Milan, Italy

**Keywords:** Percutaneous nephrolithotomy, Infectious complications, Stone, Aspiration, Safety

## Abstract

**Purpose:**

To evaluate the impact of vacuum-assisted mini-percutaneous nephrolithotomy (vamPCNL) vs. vacuum-cleaner mPCNL (vcmPCNL) on the rate of postoperative infectious complications in a cohort of patients with high risk factors for infections.

**Methods:**

We retrospectively analysed data from 145 patients who underwent mPCNL between 01/2016 and 12/2022. Patient’s demographics, stones characteristics and operative data were collected. vamPCNL and vcmPCNL were performed based on the surgeon’s preference. High-risk patients were defied as having ≥ 2 predisposing factors for infections such as a history of previous urinary tract infections, positive urine culture before surgery, stone diameter ≥ 3 cm, diabetes mellitus and hydronephrosis. Complications were graded according to modified Clavien classification. Descriptive statistics and logistic regression models were used to identify factors associated with postoperative infectious complications.

**Results:**

vamPCNL and vcmPCNL were performed in 94 (64.8%) and 51 (35.2%) cases, respectively. After surgery, infectious complications occurred in 43 (29.7%) participants. Patients who developed infectious complications had larger stone volume (*p* = 0.02) and higher rate of multiple stones (*p* = 0.01) than those who did not. Infectious complications occurred more frequently after vcmPCNL than vamPCNL (55.9% vs. 44.1%. *p* = 0.01) in high-risk patients. Longer operative time (*p* < 0.01) and length of stay (*p* < 0.01) were observed in cases with infectious complications. At multivariable logistic regression analysis, longer operative time (OR 1.1, *p* = 0.02) and vcmPCNL (OR 3.1, *p* = 0.03) procedures were independently associated with the risk of infectious complications post mPCNL, after accounting for stone volume.

**Conclusion:**

One out of three high-risk patients showed infectious complications after mPCNL. vamPCL and shorter operative time were independent protective factors for infections after surgery.

## Introduction

According to current guidelines percutaneous nephrolithotomy (PCNL) is the gold standard surgical technique for large kidney stones (> 2 cm) in adult patients [1]. Despite being considered highly effective in terms of stone free rate (SFR) [2], this procedure is not free from complications: fever, bleeding, pneumothorax, pain and sepsis are the most commonly reported in the published literature [3]. Infectious complications are frequently found after PCNL, with studies showing an incidence of systemic inflammatory response syndrome as high as 35% in patients with complex stones [4]. Furthermore, according to epidemiological data, 0.5–7.6% of patients might develop sepsis after PCNL, with serious life-threatening consequences [5, 6].

Risk factors for post-PCNL infectious complications can be related to patient’s characteristics, stone features and procedural factors [7]. Female gender, urine culture positivity, stone burden, infected stones, multiple punctures, prolonged operation time and postoperative residual stones have been previously considered as potential risk factors for infections after PCNL [7–11]. Different pathological mechanisms might explain the occurrence of these complications, such as a renal vascular damage during percutaneous access or high intrarenal pressure (IRP) during surgery, which might cause bacteria translocation into the bloodstream by pyelotubular and pyelovenous backflow [12].

Nowadays, the widespread use of miniaturized instruments in PCNL has contributed to reduce the incidence of complications while maintaining good outcomes [13, 14]. However, one of the main drawbacks of the use of small tract sizes is the increased IRP, with potential impact on infectious complications [15]. Among the newly introduced instrumentation, the vacuum-assisted access sheath has proved to reduce operative time and IRP during mini-PCNL, thus decreasing the infectious burden in a generic cohort of patients with kidney stones [16–18]. Nonetheless, it would be of clinical interest to assess if vacuum-assisted mini-PCNL (vamPCNL) could reduce the risk of infections also in patients with preoperative predisposing characteristics for post-PCNL infectious complications (such as preoperative positive urine culture, history of previous urinary tract infections, large stone volume, diabetes mellitus and hydronephrosis) [8, 19–21].

Therefore, we conducted this cross-sectional, real-life study to investigate the impact of vamPCNL, as compared to standard mPCNL, on the rate of infections complications, in a selected cohort of patients with kidney stones and high-risk factors for infections.

## Materials and methods

We performed a retrospective analysis on 209 consecutive patients who underwent mPCNL at our single tertiary-referral academic center between January 2016 and December 2022. Clinical characteristics included age, body mass index (BMI) and gender. Comorbidities were scored with the Charlson comorbidity index (CCI) [22]. The diagnosis of urolithiasis was established using preoperative urographic computerized tomography (CT) scans, which provided information on the location and density (HU) of the stones, as well as the presence of hydronephrosis. Stone volume was calculated using the ellipsoid formula (length × width × height × π × 1/6) [23]. Each patient underwent a preoperative urine culture, and if the culture was negative, a one-shot parenteral prophylaxis was administered. Patients with asymptomatic bacteriuria received targeted therapy starting 48–72 h before the procedure. In cases where patients exhibited urinary symptoms, leukocytosis, or fever, the surgery was postponed until after completion of a full course of antibiotics and a negative urine culture [9, 10].

## Surgical techniques

The surgical technique was standardized among all surgeons involved in the study. All procedures were conducted under general anesthesia with the patient positioned supine in the Valdivia position. The surgical equipment used included the MIP 16F (Karl Storz, Tuttlingen, Germany) metallic sheath and dilator (namely, vcmPCNL) [24], as well as the 16F Clear Petra disposable nephrostomic sheath (Well Lead Medical Co., Guangzhou, China) (namely, vamPCNL) [16, 17]. The Clear Petra sheath is externally plugged to prevent the medium from flowing out and it is equipped with a lateral oblique arm connected to the central vacuum system [16]. This allows the continuous aspiration of stone powder and irrigation fluid beside the scope during lithotripsy. The aspiration pressure can be regulated throughout the procedure according to surgical needs as previously reported [16]. In particular, it can be enhanced to ameliorate visibility in the presence of stone powder or blood and while withdrawing the nephroscope inside the sheath to extract stone fragments.

The equipment also included the 12F MIP nephroscope (Karl Storz, Tuttlingen, Germany) and the holmium laser (Versa-Pulse PowerSuite 100W; Lumenis, Israel).

The procedures began with retrograde pyelography to assess the pelvicaliceal anatomy and placing a ureteral catheter in the renal pelvis to inject the contrast medium. Renal puncture was performed with combined fluoroscopic and ultrasonographic guidance. Tract dilatation was done in a single step using either the MIP 16F metallic dilator or the ClearPetra sheath along with its stylet.

During the procedure, irrigation was carried out using a saline gravity bag suspended 1.5 m above the kidney level. Stone fragmentation was achieved using a 550-μm holmium:YAG laser fiber, with fragmentation settings adjusted according to the surgical requirements. Stone fragments were removed using the vacuum cleaner effect during vcmPCNL or through the aspiration-assisted sheath during vamPCNL. In cases where residual fragments could not be removed with the aforementioned devices, a flexible ureteroscope (7.9F; Olympus URF-P6, Germany) and nitinol baskets were used through the percutaneous access. As an exit strategy, an 8F nephrostomy tube was placed in all cases and left open unless major bleeding was noted, while the ureteral catheter was either left in place or removed at the end of the procedure based on the surgeon’s preference.

## Intraoperative and postoperative data

The number of access tracts, operative time (OT), defined as the time from placement of the ureteral catheter until its removal and hospital stay were recorded. According to our internal protocol, uncomplicated procedures were managed as follows: the bladder catheter was removed on postoperative day one and the nephrostomy tube was closed; on postoperative day two, a percutaneous pyelography was performed to assess ureteral canalization. If ureteral canalization was confirmed, the nephrostomy tube was removed. Patients were discharged on postoperative day three. Patients with failed antegrade ureteral canalization were managed with observation or medications (steroids) for 24–48 h and a second pyelography was performed before nephrostomy tube removal.

Postoperative complications were graded according to the PCNL-adjusted Clavien score [25, 26]. Infectious complications were defined as positive systemic inflammatory response syndrome (SIRS) criteria with bacteremia or bacteriuria, as previously reported [27].

Patients were evaluated within 3 months after surgery with a CT scan to identify residual stones [28].

Inclusion criteria: for the specific purpose of this study, we selected only patients with preoperative risk factors for infectious complications after mPCNL, such as a history of previous urinary tract infections (UTIs), preoperative indwelling ureteral catheter, positive urine culture before surgery, stone diameter ≥ 3 cm, diabetes mellitus or hydronephrosis [8, 19–21]. High-risk patients were defined as having ≥ 2 of the previously reported risk factors.

Exclusion criteria were patients with congenital renal anomalies (*N* = 10); scheduled staged procedures for large stone burden (*N* = 45); concomitant additional procedures other than PCNL (*N* = 11); endoscopic combined intrarenal surgery procedures (*N* = 2). A convenient sample of 145 patients was used for statistical analysis.

Data collection adhere to the principles of the Declaration of Helsinki. All patients signed an informed consent agreeing to share their own anonymous information for future studies. The study was approved by the Foundation IRCCS Ca’ Granda—Ospedale Maggiore Policlinico Ethical Committee (Prot. 25508).

## Statistical analysis

Distribution of data was tested with the Shapiro–Wilk test. Data are presented as medians (interquartile range; IQR) or frequencies (proportions). Descriptive statistics were used to describe the whole cohort. Second, clinical parameters, intraoperative and postoperative characteristics were compared between participants with and without postoperative infectious complications with the Mann–Whitney test and Fisher Exact Test, as indicated. Lastly, univariable and multivariable logistic regression models tested the association between clinical variables and postoperative infectious complications. Statistical analyses were performed using SPSS v.26 (IBM Corp., Armonk, NY, USA). All tests were two sided, and statistical significance level was determined at *p* < 0.05.

## Results

Table 1 details descriptive characteristics of the whole cohort and segregated according to the surgical procedure. Among the cohort of 145 patients at high risk for postoperative infections, median (IQR) age and BMI were 56 years (46–64) years and 24.5 (21.2–28.1) kg/m^2^, respectively. A CCI ≥ 1 was found in 84 (57.9%) participants. Median stone volume was 5.2 (2.1–6.9) cm^3^ and 66.2% of patients had multiple stones (Table 1). vamPCNL and vcmPCNL were performed in 94 (64.8%) and 51 (35.2%) cases, respectively. Median operative time and hospitalization time were 110 (82–144) min and 5 (4–9) days. Groups were comparable in terms of clinical and stone’s characteristics. Operative time and length of stay were shorter in the vamPCNL group compared to the vcmPCNL one (all *p* < 0.01). A JJ stent was never placed after the procedure, while only in two cases, the nephrostomy tube was closed immediately after surgery. Stone composition was similar between the two groups. After surgery, 121 (83.4%) patients were stone free and 57 (39.3%) had postoperative complications (any Clavien). Infectious complications occurred in 43 (29.7%) cases after surgery (Table 2).

**Table 1:** Demographic characteristics of the whole cohort and segregated according to the type of surgery (*n* = 145)

	Overall	vamPCNL (*N* = 94)	vcmPCNL (*N* = 51)	*p*-Value*
Age (years)				0.3
Median (IQR)	56 (46–64)	55 (46–65)	56 (46–65)	
Range	19–84	19–84	20–84	
Male gender [No. (%)]	65 (44.8)	44 (46.8)	21 (41.2)	0.5
BMI (kg/m^2^)				0.5
Median (IQR)	24.5 (21.2–28.1)	24.5 (21.1–27.8)	24.4 (22.2–28.6)	
Range	17.9–42.2	17.9–39.6	18.1–42.2	
CCI (score)				0.2
Mean (SD)	1.1 (0.4)	1.07 (0.3)	0.8 (0.1)	
Range	0–8	0–8	0–5	
CCI ≥ 1 [No. (%)]	84 (57.9)	43 (45.7)	41 (80.3)	0.1
History of UTIs [No. (%)]	27 (18.6)	19 (20.2)	8 (15.6)	0.6
Preoperative indwelling ureteral stent [No. (%)]	10 (6.8)	7 (7.4)	3 (5.8)	0.3
Preoperative positive urine culture [No. (%)]	47 (32.4)	32 (34.1)	15 (29.4)	0.5
Laterality [No. (%)]				0.7
Right	68 (46.8)	45 (47.8)	22 (43.1)	
Left	77 (53.2)	49 (52.2)	29 (56.9)	
Stone volume (cm^3^)				0.1
Median (IQR)	5.2 (2.1–6.9)	4.9 (2.0–6.8)	5.2 (2.1–6.9)	
Range	1.9–12.2	1.9–10.7	2.1–12.2	
Multiple stones [No. (%)]	96 (66.2)	63 (67.0)	33 (64.7)	0.8
Mean stone density (Hounsfield unit)				0.4
Median (IQR)	775 (600–930)	765 (590–920)	780 (610–952)	
Range	313–1563	313–1500	500–1563	
Hydronephrosis [No. (%)]	71 (48.9)	45 (47.8)	26 (50.9)	0.7
Multiple access tracts [No. (%)]	38 (26.2)	21 (22.3)	17 (33.3)	0.1
Operative time (min)				0.01
Median (IQR)	110 (82–144)	100 (75–130)	120 (100–155)	
Range	27–255	27–225	45–255	
Hospitalization time (days)				0.01
Median (IQR)	5 (4–9)	4 (3–7)	6 (5–10)	
Range	2–30	2–20	2–30	
Stone composition				0.2
CaOx mono/di-hydrate	42 (29.0)	27 (28.7)	15 (29.4)	
Ca phosphate	63 (43.4)	42 (44.6)	21 (41.1)	
Uric acid	14 (9.8)	8 (8.6)	6 (11.7)	
Cistine	3 (2.0)	2 (2.2)	1 (1.9)	
Struvite	23 (15.8)	15 (15.9)	8 (15.6)	
Postoperative complications [No. (%)]				0.6
(Highest Clavien score)				
Clavien–Dindo I–II	48 (33.1)	25 (26.5)	23 (45.0)	
Clavien–Dindo IIIa/b	9 (6.2)	4 (4.2)	5 (9.8)	
Stone free rate [No. (%)]	121 (83.4)	79 (84.0)	42 (82.3)	0.7

*p Value according to the Mann–Whitney test and Fisher Exact test

Abbreviations: *BMI* body mass index, *CCI* Charlson comorbidity index, *UTIs* urinary tract infections, *vamPCNL* vacuum-assisted mini-percutaneous nephrolithotomy, *vcmPCNL* vacuum-cleaner mini-percutaneous nephrolithotomy, *Ca* calcium, *CaOx* calcium oxalate

**Table 2:** Descriptive statistics of the cohort as segregated according to the occurrence of infectious complications (*n* = 145)

	+Infections	−Infections	*p*-Value*
Number of patients [No. (%)]	43 (29.7)	102 (70.3)	
Age (years)			0.9
Median (IQR)	54 (43–66)	56 (45–64)	
Range	19–82	24–84	
Male gender [No. (%)]	23 (53.5)	42 (41.2)	0.2
BMI (kg/m^2^)			0.5
Median (IQR)	24.8 (21.2–28.6)	24.2 (21.0–28.1)	
Range	17.9–42.2	17.7–36.1	
CCI (score)			0.3
Mean (SD)	1.1 (0.2)	0.7 (0.2)	
Range	0–8	0–6	
Laterality [No. (%)]			0.3
Right	18 (41.8)	50 (49.0)	
Left	25 (58.2)	52 (51.0)	
Stone volume (cm^3^)			0.02
Median (IQR)	5.1 (1.9–6.2)	2.3 (1.7–3.4)	
Range	2.1–12.2	1.9–8.3	
Multiple stone [No. (%)]	35 (81.3)	61 (59.8)	0.01
Staghorn stone [No. (%)]	25 (58.1)	29 (28.4)	0.02
Mean stone density (Hounsfield unit)			0.04
Median (IQR)	676 (553–906)	825 (645–1004)	
Range	313–1096	392–1563	
Multiple access tracts [No. (%)]	16 (37.2)	22 (21.5)	0.06
Procedure type [No. (%)]			0.01
vamPCNL	19 (44.1)	75 (73.5)	
vcmPCNL	24 (55.9)	27 (26.5)	
Operative time (min)			< 0.01
Median (IQR)	142 (116–174)	100 (74–136)	
Range	50–255	27–234	
Hospitalization time (days)			< 0.01
Median (IQR)	7 (6–12)	4 (3–6)	
Range	4–30	2–18	
Stone composition			0.2
CaOx mono/di-hydrate	8 (18.7)	34 (33.3)	
Ca phosphate	19 (44.2)	44 (43.2)	
Uric acid	6 (13.9)	8 (7.8)	
Cistine	1 (2.3)	2 (1.9)	
Struvite	9 (20.9)	14 (13.8)	
Stone free rate [No. (%)]	30 (69.7)	91 (89.2)	0.04

Abbreviations: *BMI* body mass index, *CCI* Charlson comorbidity index, *vamPCNL* vacuum-assisted mini-percutaneous nephrolithotomy, *vcmPCNL* vacuum-cleaner mini-percutaneous nephrolithotomy, *Ca* calcium, *CaOx* calcium oxalate

^*^p Value according to the Mann–Whitney test and Fisher Exact test, as indicated

Patients who developed infectious complications had larger stone volume [5.1 (1.9–6.2) cm^3^ vs. 2.3 (1.7–3.4) cm^3^, *p* = 0.02] and higher rate of multiple (81.3% vs. 59.8%, *p* = 0.01) and staghorn stones (58.1% vs. 28.4%, *p* = 0.02) than those who did not (Table 2). Infectious complications occurred more frequently after vcmPCNL than vamPCNL (55.9% vs. 44.1%. *p* = 0.01) in this cohort of high-risk patients. Longer operative time [142 (116–174) min vs. 100 (74–136) min, *p* < 0.01] and length of stay [7 (6–12) days vs. 4 (3–6) days, *p* < 0.01] along with lower rate of stone free status (69.7% vs. 89.2%, *p* = 0.04) were observed in cases with infectious complications (Table 2). Stone composition was not associated with postoperative infectious complications.

Table 3 reports logistic regression models testing potential predictors for infections complications after surgery. At univariable analysis, stone volume (OR 1.2, CI 1.02–1.42, *p* = 0.03), stone density (OR 0.97, CI 0.96–0.99, *p* = 0.01), operative time (OR 1.1, CI 1.01–1.34, *p* = 0.02) and vcmPCNL procedures (OR 3.5, CI 1.66–7.39, *p* = 0.001) were associated with infections complications. At multivariable logistic regression analysis, longer operative time (OR 1.1, CI 1.01–1.26, *p* = 0.02) and vcmPCNL (OR 3.1, CI 1.07–9.11, *p* = 0.03) procedures were independently associated with the risk of infectious complications post-mPCNL, after accounting for stone volume.

**Table 3:** Logistic regression models predicting infectious complications

	UVA model			MVA model		
	OR	*p*-Value	95% CI	OR	*p*-Value	95% CI
Age	0.98	0.9	0.97–1.02			
BMI	1.1	0.2	0.97–1.12			
CCI ≥ 1	1.2	0.3	0.87–1.42			
Stone volume	1.2	0.03	1.02–1.42	1.1	0.4	0.97–1.21
Stone density	0.97	0.01	0.96–0.99			
vcmPCNL vs. vamPCNL	3.5	0.001	1.66–7.39	3.1	0.03	1.07–9.11
Operative time	1.1	0.02	1.01–1.34	1.1	0.02	1.01–1.26
Infectious stone	1.5	0.2	0.70–3.25			

Abbreviations: *UVA* univariate model, *MVA* multivariate model, *BMI* body mass index, *CHI* Charlson comorbidity index; *vamPCNL* vacuum-assisted mini-percutaneous nephrolithotomy; *vcmPCNL* vacuum-cleaner mini-percutaneous nephrolithotomy

## Discussion

In this study, we aimed to investigate potential risk factors for infectious complications in patients with kidney stones and preoperative clinical characteristics connected to post-PCNL infection. We found that, in this specific group, one out of three patients developed postoperative infections. Of clinical importance, we revealed that shorter operative time and vacuum assisted procedures were associated with a lower risk of infectious complications after mPCNL in high-risk patients.

Our project was motivated by the lack of studies specifically looking at factors associated with post-PCNL infections in high-risk patients. Indeed, from an epidemiological point of view, the rate of patients with kidney stones at high risk for postoperative infections is expected to increase in future years for several reasons. First, the prevalence of stone disease rises worldwide and a growing number of patients will need a minimally invasive procedure to treat kidney stones [29]; second, recurrent urinary tract infections and related antibiotic treatment are frequent and serious issues in the everyday clinical practice, thus predisposing patients to further infective danger [30, 31]; third, also connected to the previous observation, the rate of infected stones (e.g. struvite) in increasing, at least in Western countries [32]; forth, continued improvements in life expectancy have seen the growth of the elderly population, with associated chronic diseases (e.g. diabetes mellitus, immune disorders) [33]. Thus, an increase in the number of elderly and comorbid patients with renal stones is to be expected. Consequently, mPCNL will be more frequently performed in this population in future, with potential risk for infectious complications related to their lower overall health condition [34, 35].

Infections are among the most common adverse events after PCNL with an incidence ranging from 2.4 to 40.4% according to a recent systematic review [8]. From a pathophysiological standpoint, post-PCNL infections can be caused by bacterial presence in stone or renal pelvic urine, which enters the bloodstream during stone manipulation through pyelovenous, pyelolymphatic, pyelotubular backflows and forniceal rupture [36]. The two components of bacteremia during PCNL are stone colonization by bacteria and the release of endotoxin as lipopolysaccharide during stone fragmentation and continuous fluid irrigation by small vein and lymphatic channels [36]. Moreover, high levels of intrapelvic pressure during surgery can cause pyelovenous and pyelolymphatic backflow or even rupture of the collecting system, possibly leading to peri-renal hematoma or urosepsis [37].

For such reasons, several clinical periprocedural factors have been associated with an increased risk of post-PCNL infections. Preoperative patient’s risk characteristics include age, diabetes mellitus, positive urine culture and stone size [8, 19–21]. Intraoperative factors associated with post-PCNL infections are procedural tract size, renal pelvic pressure and operative time [11, 15, 36].

It is expected that, as the size of the stones increases, urinary obstruction also increases, as does the difficulty of the procedure and operative time [38]. Moreover, longer surgical time results in sustained high pressure within the renal pelvis, contributing to bacterial spread and risk of postoperative infections [39]. Intrarenal pressure can be effectively reduced by using a suction-assisted device during mPCNL [40]. In a previous study, Zanetti et al. showed that mean IRP was always lower than the threshold of pyelovenous backflow and the accumulative time with IRP over this limit was short during vamPCNL [16]. Similarly, Lievore et al. analysed a series of men with kidney stones and found that vamPCNL reduced operative time and the risk of postoperative infections compared to vcmPCNL [17]. The advantage of vamPCNL was also highlighted in terms of hospitalization costs [18]. However, the protective role of vamPCNL has never been investigated in stone patients with high risk for infections.

In this study, we confirmed that vamPCNL was associated with shorter operative time than vcmPCNL and that vamPCNL procedures reduced the risk of infectious complications even in patients at high risk for infections. In particular, patients treated with non-suctioning mPCNL had a three-times higher risk of infections compared to those treated with vamPCNL. This result is achieved through the continuous suction system of vamPCNL, which allows for shorter surgical procedure times and, simultaneously, working at lower intrarenal pressure. Therefore, vamPCNL appears to be the ideal surgical technique also for patients at high risk of developing infectious complications. From a clinical standpoint, lowering the rate of postoperative infectious complications will lead to shorter hospitalization time, lower impact on patient’s health and reducing hospitalization costs.

This study is innovative since it is the first to evaluate the prevalence and predictive factors for infectious complications in a homogenous cohort of high-risk patients for infections after mPCNL, confirming the effectives of vamPCNL in reducing this risk. Moreover, our results are relevant from the everyday clinical practice, where high-risk patients are increasing and the identification of the best surgical approach to reduce postoperative complication is of primary importance.

Limitations of this study are the single centre-based and retrospective design study, which raises the possibility of selection biases. Thereof, larger prospective studies across different centres and cohorts are needed to externally validate our findings. The timing from stone fragmentation to endoscopic stone-free was not recorded, therefore, we could not compare surgical efficiency between vamPCNL and vcmPCNL. Lastly, high-risk patients were defined as the presence of ≥ 2 risk factors, which is an arbitrary definition yet based on published data on predisposing characteristics for infectious complications [8, 19–21].

## Conclusion

In a cohort of patients with kidney stones and high-risk factors for infections, approximately 30% of participants developed infectious complications after mPCNL. vamPCL and shorter operative time were independent protective factors for infections after surgery. vamPCNL confirmed to be associated with lower infectious complications even in high-risk patients.

## Data Availability

Data is available upon request to the corresponding author.
